# (2,2′-Bi­pyridine-κ^2^*N*,*N*′)(4,4′-dimeth­oxy-2,2′-bipyridine-κ^2^*N*,*N*′)palladium(II) bis­(tri­fluoro­meth­anesulfonate)

**DOI:** 10.1107/S2414314624001093

**Published:** 2024-02-08

**Authors:** Brittney B. Vargas, Hadi D. Arman, Rafael A. Adrian

**Affiliations:** a Department of Chemistry and Biochemistry, University of the Incarnate Word, San Antonio, Texas 78209, USA; b Department of Chemistry The University of Texas at San Antonio,San Antonio Texas 78249 USA; Vienna University of Technology, Austria

**Keywords:** crystal structure, palladium(II) complex, square-planar coordination environment, 2,2′-bi­pyridine, 4,4′-dimeth­oxy-2,2′-bi­pyridine, tri­fluoro­methane­sulfonate salt, τ_4_ descriptor

## Abstract

In the title complex salt, [Pd(C_10_H_8_N_2_)(C_12_H_12_N_2_O_2_)](CF_3_SO_3_)_2_, the palladium(II) atom is fourfold coordinated by two chelating ligands, 2,2′-bi­pyridine and 4,4′-dimeth­oxy-2,2′-bi­pyridine, in a distorted square-planar environment. In the crystal, weak π–π stacking inter­actions between the 2,2′-bi­pyridine rings [centroid-to-centroid distances = 3.8984 (19) Å] and between the 4,4′-dimeth­oxy-2,2′-bi­pyridine rings [centroid-to-centroid distances = 3.747 (18) Å] contribute to the alignment of the complex cations in columns parallel to the *b*-axis direction.

## Abstract 2

In the cation of the title complex, the central palladium(II) atom is surrounded by two bidentate ligands, 2,2′-bi­pyridine and 4,4′-dimeth­oxy-2,2′-bi­pyridine, in a distorted square-planar environment.

## Graphical abstract

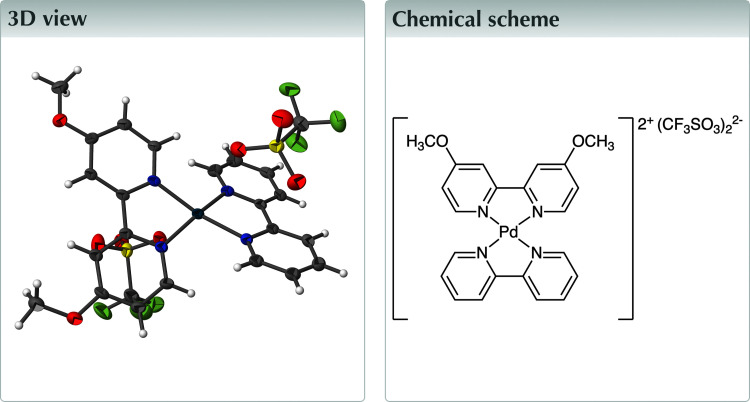

## Structure description

Bi­pyridine derivatives continue to be recognized as valuable ligands for the synthesis of new transition-metal complexes, including cobalt(II) (Kondori *et al.*, 2021 ▸), ruthenium(II) (Benson *et al.*, 2021 ▸; Maier *et al.*, 2022 ▸), iron(II) (Karges & Gasser, 2020 ▸), copper(II) (Shchegolkov *et al.*, 2021 ▸) and palladium(II) (Komlyagina *et al.*, 2023 ▸) to mention a few. Recently, palladium(II) complexes containing 2,2′-bi­pyridine as ligand have shown significant cytotoxicity against HT-29 (colorectal adenocarcinoma), MCF-7 (breast), and HeLa (human squamous cervical adenocarcinoma) cancer cell lines (Tabrizi *et al.*, 2020 ▸). As part of our research in this area, we describe herein the synthesis and structure of the title palladium(II) complex.

**Figure 1:**
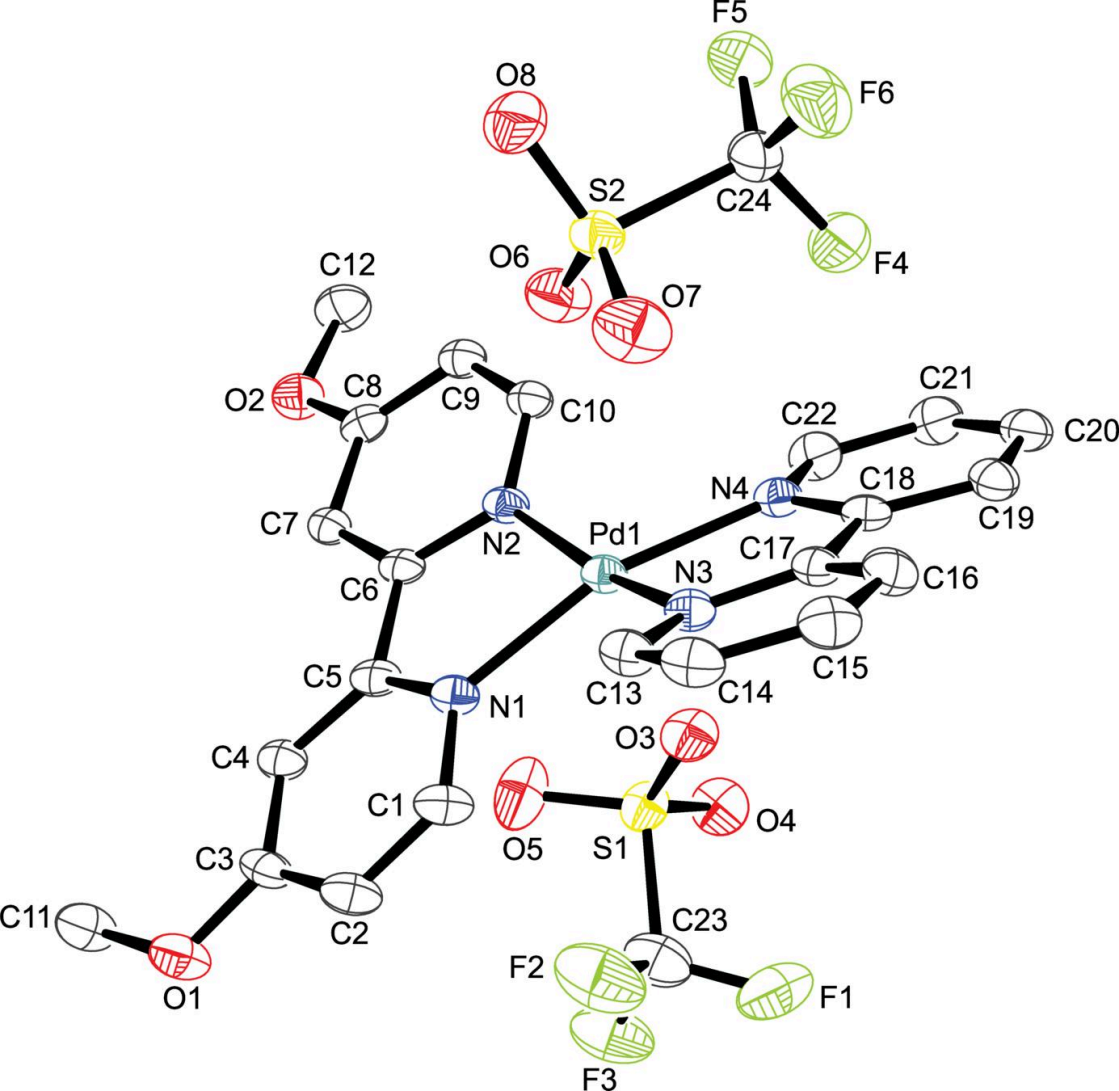
The structures of the mol­ecular entities present in title compound with displacement ellipsoids drawn at the 50% probability level; H atoms are omitted for clarity.

The asymmetric unit comprises one complex cation and two tri­fluoro­methane­sulfonate anions. The palladium(II) atom shows a distorted square-planar coordination environment defined by a bidentate 2,2′-bi­pyridine ligand and a bidentate 4,4′-dimeth­oxy-2,2′-bi­pyridine; tri­fluoro­methane­sulfonate ions sit in the outer coordination sphere, balancing the charge of the complex metal cation (Fig. 1 ▸). The Pd—N bond lengths are in good agreement with those in comparable square-planar 4,4′-dimeth­oxy-2,2′-bi­pyridine palladium(II) complexes currently available in the Cambridge Structural Database (CSD, version 5.45, Nov 2023; Groom *et al.*, 2016 ▸): refcodes BEPVIF (Yang *et al.*, 2022 ▸), WISQUO (Komlyagina *et al.*, 2023 ▸); WISRAV (Komlyagina *et al.*, 2023 ▸). The τ_4_ descriptor value (Yang *et al.*, 2007 ▸) of 0.22 reflects a significant distortion from a perfect square-planar coordin­ation (τ_4_ = 0). Numerical data for relevant bond lengths and angles are presented in Table 1 ▸.

**Figure 2:**
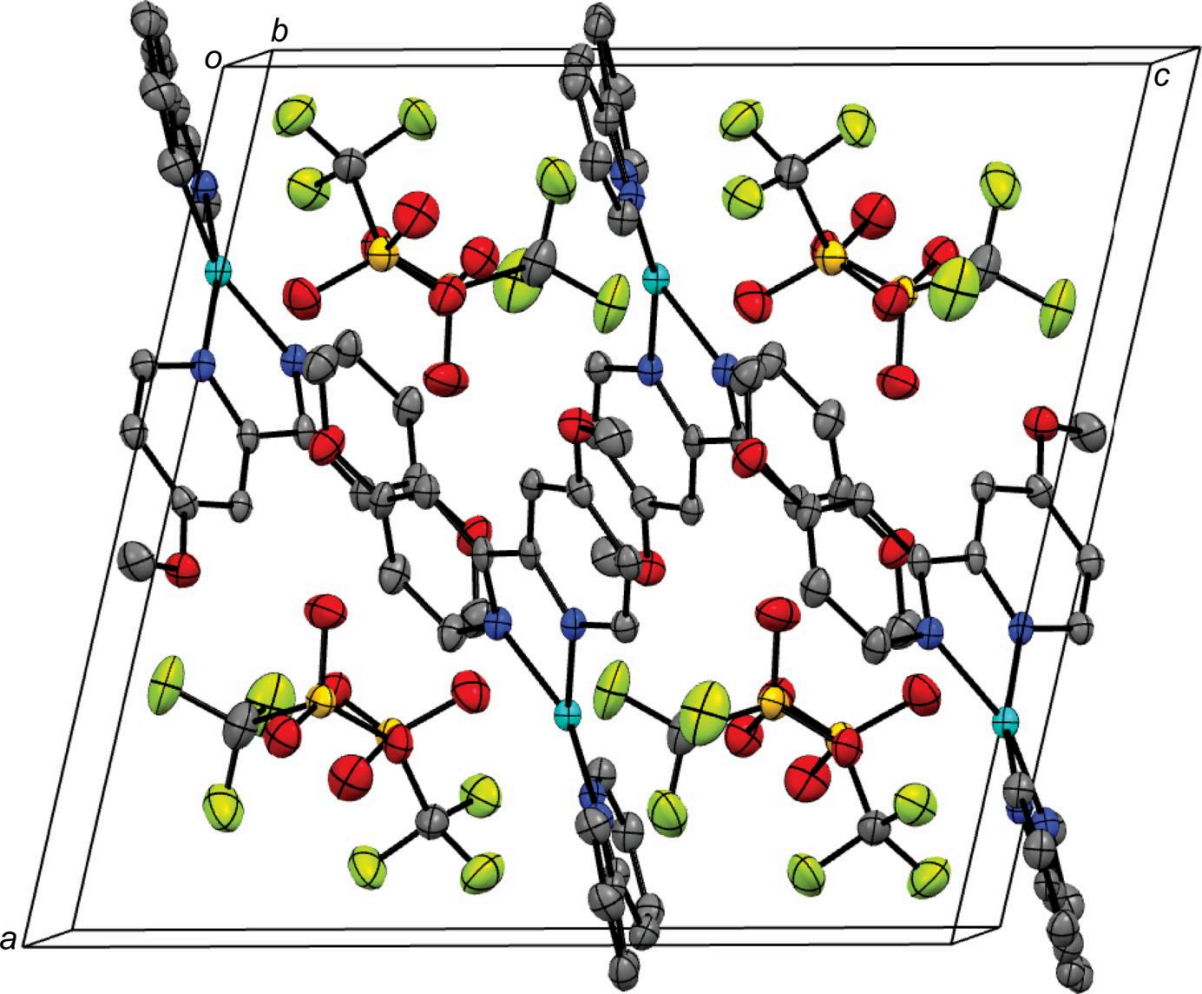
Perspective view of the crystal packing of the title complex approximately along the *b* axis; H atoms are omitted for clarity.

**Figure 3:**
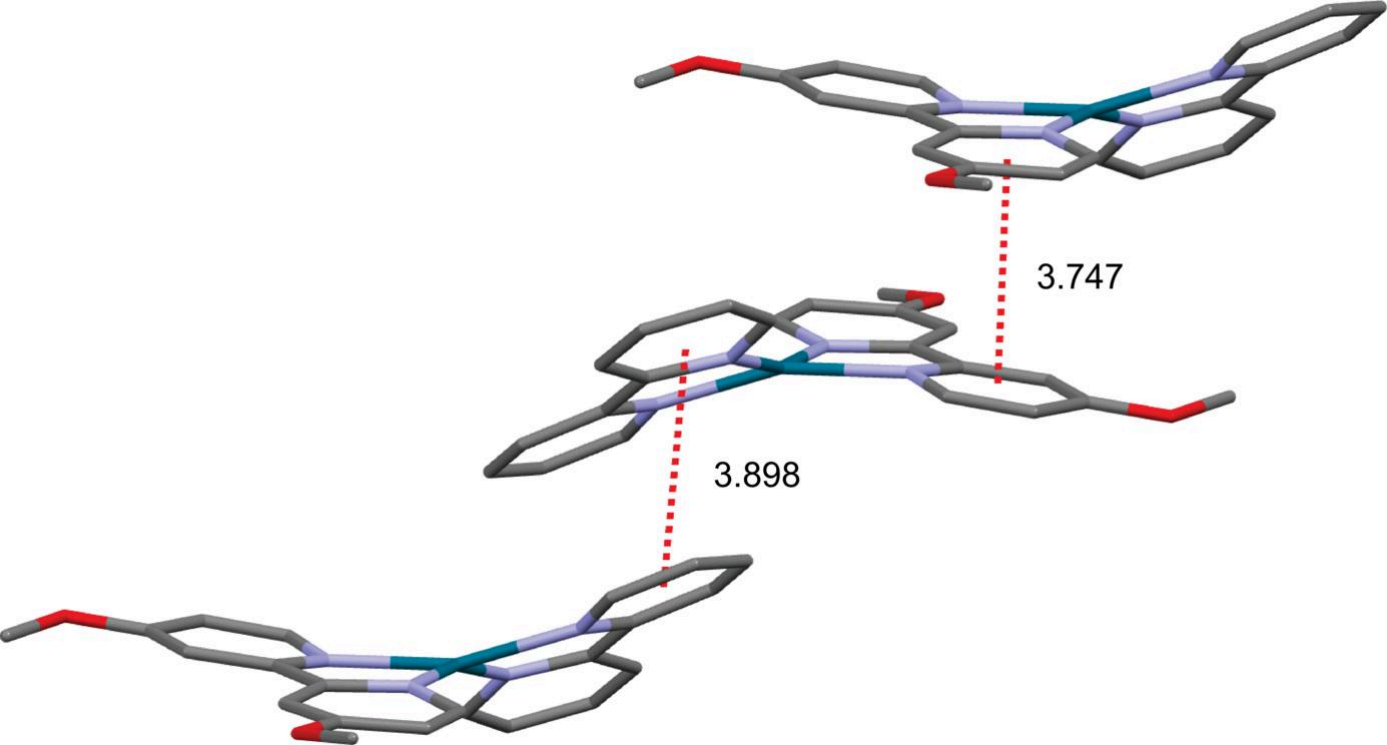
Capped sticks representation of the complex cation showing π–π stacking inter­actions (red). H atoms and anions are omitted for clarity.

In the extended structure, the complex packs into columns extending parallel to the *b* axis (Fig. 2 ▸). Contiguous pyridine rings show weak π–π stacking inter­actions, with centroid-to-centroid distances (*Cg*⋯*Cg*) alternating between 3.7472 (18) Å (between 4,4′-dimeth­oxy-2,2′-bi­pyridine ligands) and 3.8984 (19) Å (between 2,2′-bi­pyridine ligands), and offset distances of 1.641 and 1.769 Å, respectively (Fig. 3 ▸). No other significant supra­molecular inter­actions are present in the crystal packing of the title compound.

## Synthesis and crystallization

The title complex was prepared by adding Ag(CF_3_SO_3_) (0.0771 g, 0.300 mmol) to an aceto­nitrile suspension (40 ml) of [Pd(2,2′-bi­pyridine)Cl_2_] (0.100 g, 0.300 mmol). The mixture was heated, with stirring, at 333 K for 2 h and then filtered using a PTFE syringe filter to remove the precipitated AgCl. 4,4′-Dimeth­oxy-2,2′-bi­pyridine (0.0649 g, 0.300 mmol) was added to the resulting solution and heated at 343 K to reduce the volume of the solution to 10 ml. X-ray diffraction quality crystals of the title complex were obtained by vapor diffusion of ether over the resulting concentrated aceto­nitrile solution.

## Refinement

Crystal data, data collection and structure refinement details are summarized in Table 2 ▸.

## Supplementary Material

Crystal structure: contains datablock(s) I, global. DOI: 10.1107/S2414314624001093/wm4206sup1.cif

Structure factors: contains datablock(s) I. DOI: 10.1107/S2414314624001093/wm4206Isup2.hkl

Supporting information file. DOI: 10.1107/S2414314624001093/wm4206Isup3.mol

CCDC reference: 2330055

Additional supporting information:  crystallographic information; 3D view; checkCIF report

## Figures and Tables

**Table 1:** Selected geometric parameters (Å, °)

Pd1—N2	2.022 (3)	Pd1—N3	2.033 (3)
Pd1—N1	2.029 (3)	Pd1—N4	2.046 (3)
			
N2—Pd1—N1	79.63 (10)	N2—Pd1—N4	101.76 (10)
N2—Pd1—N3	165.80 (10)	N1—Pd1—N4	162.05 (10)
N1—Pd1—N3	102.93 (10)	N3—Pd1—N4	80.18 (10)

**Table 2:** Experimental details

Crystal data	
Chemical formula	[Pd(C_10_H_8_N_2_)(C_12_H_12_N_2_O_2_)](CF_3_SO_3_)_2_
*M*_r_	776.96
Crystal system, space group	Monoclinic, *P*2_1_/*c*
Temperature (K)	100
*a*, *b*, *c* (Å)	14.4340 (3), 13.9644 (2), 14.2126 (2)
β (°)	102.5361 (16)
*V* (Å^3^)	2796.41 (8)
*Z*	4
Radiation type	Cu *K*α
μ (mm^−1^)	7.64
Crystal size (mm)	0.23 × 0.11 × 0.08

Data collection	
Diffractometer	XtaLAB Synergy, Dualflex, HyPix
Absorption correction	Gaussian (*CrysAlis PRO*; Rigaku OD, 2022 ▸)
*T*_min_, *T*_max_	0.512, 1.000
No. of measured, independent and observed [*I* > 2σ(*I*)] reflections	26480, 5585, 5101
*R*_int_	0.053
(sin θ/λ)_max_ (Å^−1^)	0.630

Refinement	
*R*[*F*^2^ > 2σ(*F*^2^)], *wR*(*F*^2^), *S*	0.038, 0.100, 1.04
No. of reflections	5585
No. of parameters	408
H-atom treatment	H-atom parameters constrained
Δρ_max_, Δρ_min_ (e Å^−3^)	0.80, −1.09

Computer programs: *CrysAlis PRO* (Rigaku OD, 2022 ▸), *SHELXT* (Sheldrick, 2015*a* ▸), *SHELXL* (Sheldrick, 2015*b* ▸), and *OLEX2* (Dolomanov *et al.*, 2009 ▸).

## full crystallographic data

### (2,2'-Bipyridine-κ2N,N')(4,4'-dimethoxy-2,2'-bipyridine-κ2N,N')palladium(II) bis(trifluoromethanesulfonate) Crystal data

**Table:**

[Pd(C_10_H_8_N_2_)(C_12_H_12_N_2_O_2_)](CF_3_SO_3_)_2_	*F*(000) = 1552
*M**_r_* = 776.96	*D*_x_ = 1.845 Mg m^−^^3^
Monoclinic, *P*2_1_/*c*	Cu *K*α radiation, λ = 1.54184 Å
*a* = 14.4340 (3) Å	Cell parameters from 13876 reflections
*b* = 13.9644 (2) Å	θ = 4.4–75.4°
*c* = 14.2126 (2) Å	µ = 7.64 mm^−^^1^
β = 102.5361 (16)°	*T* = 100 K
*V* = 2796.41 (8) Å^3^	Block, yellow
*Z* = 4	0.23 × 0.11 × 0.08 mm

### (2,2'-Bipyridine-κ2N,N')(4,4'-dimethoxy-2,2'-bipyridine-κ2N,N')palladium(II) bis(trifluoromethanesulfonate) Data collection

**Table:**

XtaLAB Synergy, Dualflex, HyPix diffractometer	5585 independent reflections
Radiation source: micro-focus sealed X-ray tube, PhotonJet (Cu) X-ray Source	5101 reflections with *I* > 2σ(*I*)
Mirror monochromator	*R*_int_ = 0.053
Detector resolution: 10.0000 pixels mm^-1^	θ_max_ = 76.2°, θ_min_ = 3.1°
ω scans	*h* = −17→18
Absorption correction: gaussian (CrysAlisPro; Rigaku OD, 2022)	*k* = −17→16
*T*_min_ = 0.512, *T*_max_ = 1.000	*l* = −15→17
26480 measured reflections	

### (2,2'-Bipyridine-κ2N,N')(4,4'-dimethoxy-2,2'-bipyridine-κ2N,N')palladium(II) bis(trifluoromethanesulfonate) Refinement

**Table:**

Refinement on *F*^2^	Primary atom site location: dual
Least-squares matrix: full	Hydrogen site location: inferred from neighbouring sites
*R*[*F*^2^ > 2σ(*F*^2^)] = 0.038	H-atom parameters constrained
*wR*(*F*^2^) = 0.100	*w* = 1/[σ^2^(*F*_o_^2^) + (0.049*P*)^2^ + 5.4251*P*] where *P* = (*F*_o_^2^ + 2*F*_c_^2^)/3
*S* = 1.04	(Δ/σ)_max_ = 0.001
5585 reflections	Δρ_max_ = 0.80 e Å^−^^3^
408 parameters	Δρ_min_ = −1.09 e Å^−^^3^
0 restraints	

### (2,2'-Bipyridine-κ2N,N')(4,4'-dimethoxy-2,2'-bipyridine-κ2N,N')palladium(II) bis(trifluoromethanesulfonate) Special details

**Table:**

Geometry. All esds (except the esd in the dihedral angle between two l.s. planes) are estimated using the full covariance matrix. The cell esds are taken into account individually in the estimation of esds in distances, angles and torsion angles; correlations between esds in cell parameters are only used when they are defined by crystal symmetry. An approximate (isotropic) treatment of cell esds is used for estimating esds involving l.s. planes.

### (2,2'-Bipyridine-κ2N,N')(4,4'-dimethoxy-2,2'-bipyridine-κ2N,N')palladium(II) bis(trifluoromethanesulfonate) Fractional atomic coordinates and isotropic or equivalent isotropic displacement parameters (Å^2^)

**Table:**

	*x*	*y*	*z*	*U*_iso_*/*U*_eq_	
Pd1	0.25207 (2)	0.48236 (2)	0.49500 (2)	0.02008 (9)	
S2	0.22124 (6)	0.40289 (6)	0.19795 (6)	0.02732 (18)	
S1	0.27163 (6)	0.63092 (6)	0.75710 (6)	0.02833 (18)	
F5	0.15001 (17)	0.55231 (18)	0.09497 (18)	0.0472 (6)	
O2	0.58599 (17)	0.76062 (17)	0.54340 (17)	0.0310 (5)	
O3	0.22186 (18)	0.59380 (19)	0.66506 (17)	0.0351 (6)	
F6	0.06435 (18)	0.4276 (2)	0.06797 (18)	0.0533 (6)	
F4	0.06969 (19)	0.5071 (2)	0.19721 (18)	0.0548 (7)	
O6	0.2702 (2)	0.4622 (2)	0.27515 (19)	0.0406 (6)	
F1	0.1486 (2)	0.5453 (2)	0.84015 (18)	0.0586 (7)	
O1	0.55650 (19)	0.32506 (19)	0.82842 (18)	0.0365 (6)	
F3	0.2887 (2)	0.5636 (2)	0.93124 (17)	0.0641 (8)	
O7	0.1755 (2)	0.3204 (2)	0.2284 (2)	0.0481 (7)	
O5	0.37298 (19)	0.6267 (2)	0.7740 (2)	0.0495 (8)	
N1	0.35491 (19)	0.41902 (19)	0.59584 (18)	0.0224 (5)	
N3	0.15739 (19)	0.37451 (19)	0.45377 (18)	0.0222 (5)	
O8	0.2716 (2)	0.3841 (2)	0.1230 (2)	0.0430 (7)	
O4	0.2332 (2)	0.71853 (19)	0.78532 (19)	0.0389 (6)	
F2	0.2640 (3)	0.4555 (2)	0.8203 (2)	0.0700 (9)	
N4	0.13534 (19)	0.56042 (19)	0.43114 (18)	0.0229 (5)	
N2	0.35692 (19)	0.58047 (18)	0.50606 (17)	0.0212 (5)	
C5	0.4350 (2)	0.4719 (2)	0.6269 (2)	0.0220 (6)	
C17	0.0694 (2)	0.4035 (2)	0.4067 (2)	0.0222 (6)	
C1	0.3429 (3)	0.3403 (2)	0.6465 (2)	0.0285 (7)	
H1	0.285816	0.304663	0.627345	0.034*	
C13	0.1788 (2)	0.2810 (2)	0.4545 (2)	0.0259 (7)	
H13	0.241428	0.261207	0.483592	0.031*	
C8	0.5106 (2)	0.7026 (2)	0.5260 (2)	0.0225 (6)	
C19	−0.0320 (2)	0.5509 (2)	0.3674 (2)	0.0256 (7)	
H19	−0.087525	0.513068	0.347393	0.031*	
C4	0.5058 (2)	0.4444 (2)	0.7039 (2)	0.0237 (6)	
H4	0.561670	0.481893	0.722918	0.028*	
C6	0.4383 (2)	0.5611 (2)	0.5720 (2)	0.0204 (6)	
C9	0.4295 (2)	0.7186 (2)	0.4548 (2)	0.0244 (6)	
H9	0.425302	0.772082	0.412835	0.029*	
C10	0.3557 (2)	0.6550 (2)	0.4466 (2)	0.0227 (6)	
H10	0.301173	0.664352	0.396410	0.027*	
C22	0.1284 (3)	0.6560 (2)	0.4315 (2)	0.0293 (7)	
H22	0.183625	0.692736	0.456607	0.035*	
C14	0.1131 (3)	0.2125 (2)	0.4144 (2)	0.0296 (7)	
H14	0.130078	0.146652	0.416703	0.036*	
C15	0.0224 (3)	0.2410 (3)	0.3710 (2)	0.0307 (7)	
H15	−0.024666	0.194961	0.345058	0.037*	
C18	0.0555 (2)	0.5074 (2)	0.4013 (2)	0.0230 (6)	
C3	0.4941 (3)	0.3607 (2)	0.7530 (2)	0.0268 (7)	
C16	0.0009 (2)	0.3377 (3)	0.3657 (2)	0.0283 (7)	
H16	−0.060524	0.358768	0.334111	0.034*	
C20	−0.0376 (3)	0.6500 (3)	0.3631 (2)	0.0302 (7)	
H20	−0.096400	0.680803	0.337457	0.036*	
C7	0.5165 (2)	0.6199 (2)	0.5829 (2)	0.0201 (6)	
H7	0.573299	0.604693	0.628131	0.024*	
C2	0.4095 (3)	0.3096 (2)	0.7245 (2)	0.0303 (7)	
H2	0.398274	0.253950	0.758903	0.036*	
C24	0.1222 (3)	0.4770 (3)	0.1370 (3)	0.0334 (8)	
C21	0.0431 (3)	0.7033 (3)	0.3965 (3)	0.0322 (7)	
H21	0.040546	0.771242	0.395580	0.039*	
C12	0.5745 (3)	0.8532 (3)	0.4959 (3)	0.0364 (8)	
H12A	0.556581	0.844023	0.425981	0.055*	
H12B	0.634444	0.888665	0.512401	0.055*	
H12C	0.524744	0.889479	0.517390	0.055*	
C11	0.6492 (3)	0.3687 (3)	0.8529 (3)	0.0374 (8)	
H11A	0.677878	0.368095	0.796451	0.056*	
H11B	0.689463	0.332793	0.905511	0.056*	
H11C	0.643101	0.435020	0.873438	0.056*	
C23	0.2422 (3)	0.5447 (3)	0.8415 (3)	0.0438 (10)	

### (2,2'-Bipyridine-κ2N,N')(4,4'-dimethoxy-2,2'-bipyridine-κ2N,N')palladium(II) bis(trifluoromethanesulfonate) Atomic displacement parameters (Å^2^)

**Table:**

	*U*^11^	*U*^22^	*U*^33^	*U*^12^	*U*^13^	*U*^23^
Pd1	0.02596 (14)	0.01770 (14)	0.01692 (13)	0.00027 (8)	0.00541 (9)	−0.00010 (8)
S2	0.0326 (4)	0.0236 (4)	0.0260 (4)	0.0005 (3)	0.0068 (3)	0.0021 (3)
S1	0.0303 (4)	0.0315 (4)	0.0237 (4)	−0.0033 (3)	0.0070 (3)	−0.0117 (3)
F5	0.0499 (13)	0.0452 (14)	0.0478 (13)	0.0092 (11)	0.0134 (11)	0.0216 (11)
O2	0.0348 (13)	0.0276 (12)	0.0310 (12)	−0.0050 (10)	0.0080 (10)	0.0008 (10)
O3	0.0414 (14)	0.0410 (14)	0.0232 (11)	−0.0027 (11)	0.0075 (10)	−0.0128 (10)
F6	0.0484 (14)	0.0636 (17)	0.0412 (13)	0.0002 (12)	−0.0051 (10)	−0.0074 (12)
F4	0.0526 (15)	0.0778 (18)	0.0388 (13)	0.0264 (13)	0.0206 (11)	0.0045 (12)
O6	0.0447 (15)	0.0442 (15)	0.0308 (13)	−0.0057 (12)	0.0033 (11)	0.0030 (12)
F1	0.0636 (17)	0.0736 (18)	0.0437 (14)	−0.0250 (14)	0.0230 (12)	−0.0032 (13)
O1	0.0430 (14)	0.0343 (14)	0.0292 (12)	−0.0049 (11)	0.0012 (11)	0.0096 (11)
F3	0.093 (2)	0.0685 (19)	0.0246 (11)	−0.0018 (16)	−0.0006 (12)	−0.0001 (12)
O7	0.0592 (18)	0.0342 (15)	0.0493 (16)	−0.0108 (13)	0.0081 (14)	0.0058 (13)
O5	0.0331 (14)	0.067 (2)	0.0491 (16)	−0.0068 (13)	0.0096 (12)	−0.0278 (15)
N1	0.0283 (13)	0.0210 (13)	0.0178 (12)	−0.0009 (10)	0.0050 (10)	0.0000 (10)
N3	0.0279 (13)	0.0211 (13)	0.0176 (12)	−0.0021 (10)	0.0050 (10)	−0.0019 (10)
O8	0.0493 (16)	0.0425 (16)	0.0413 (15)	0.0132 (13)	0.0187 (12)	0.0002 (12)
O4	0.0479 (15)	0.0321 (13)	0.0366 (14)	0.0009 (12)	0.0088 (12)	−0.0113 (11)
F2	0.111 (3)	0.0356 (14)	0.0587 (17)	0.0080 (15)	0.0078 (17)	0.0039 (13)
N4	0.0291 (13)	0.0217 (13)	0.0193 (12)	0.0016 (11)	0.0082 (10)	0.0010 (10)
N2	0.0288 (13)	0.0197 (12)	0.0157 (11)	0.0025 (10)	0.0062 (10)	−0.0002 (10)
C5	0.0340 (17)	0.0185 (15)	0.0151 (13)	0.0003 (12)	0.0085 (12)	−0.0030 (11)
C17	0.0281 (15)	0.0228 (15)	0.0160 (13)	0.0010 (12)	0.0058 (11)	0.0000 (11)
C1	0.0384 (18)	0.0258 (17)	0.0212 (15)	−0.0060 (14)	0.0059 (13)	0.0032 (13)
C13	0.0351 (17)	0.0197 (15)	0.0229 (15)	0.0018 (13)	0.0064 (13)	0.0001 (12)
C8	0.0302 (16)	0.0176 (14)	0.0223 (14)	−0.0023 (12)	0.0116 (12)	−0.0036 (12)
C19	0.0298 (16)	0.0282 (17)	0.0185 (14)	0.0019 (13)	0.0044 (12)	0.0004 (12)
C4	0.0317 (16)	0.0221 (16)	0.0176 (13)	0.0005 (13)	0.0062 (12)	−0.0020 (12)
C6	0.0310 (16)	0.0178 (15)	0.0138 (13)	0.0037 (12)	0.0084 (11)	−0.0024 (11)
C9	0.0338 (17)	0.0200 (15)	0.0211 (14)	0.0035 (13)	0.0099 (12)	0.0001 (12)
C10	0.0287 (16)	0.0204 (15)	0.0197 (14)	0.0022 (12)	0.0069 (12)	0.0027 (12)
C22	0.0372 (18)	0.0207 (16)	0.0321 (17)	0.0019 (13)	0.0120 (14)	−0.0003 (13)
C14	0.0432 (19)	0.0212 (16)	0.0246 (16)	−0.0003 (14)	0.0078 (14)	−0.0006 (13)
C15	0.0383 (18)	0.0289 (18)	0.0245 (16)	−0.0066 (14)	0.0058 (14)	−0.0040 (14)
C18	0.0296 (16)	0.0261 (16)	0.0139 (13)	−0.0006 (13)	0.0055 (12)	0.0008 (11)
C3	0.0370 (17)	0.0268 (17)	0.0153 (13)	0.0049 (14)	0.0028 (12)	0.0029 (12)
C16	0.0319 (17)	0.0310 (17)	0.0204 (14)	−0.0015 (14)	0.0018 (12)	−0.0003 (13)
C20	0.0378 (18)	0.0318 (18)	0.0224 (15)	0.0098 (14)	0.0095 (13)	0.0049 (13)
C7	0.0271 (15)	0.0184 (14)	0.0152 (13)	0.0030 (12)	0.0056 (11)	−0.0028 (11)
C2	0.0430 (19)	0.0228 (16)	0.0240 (15)	−0.0051 (14)	0.0047 (14)	0.0049 (13)
C24	0.0339 (18)	0.040 (2)	0.0260 (17)	0.0054 (15)	0.0059 (14)	−0.0004 (15)
C21	0.041 (2)	0.0244 (17)	0.0346 (18)	0.0065 (14)	0.0156 (15)	0.0030 (14)
C12	0.044 (2)	0.0284 (19)	0.0395 (19)	−0.0049 (15)	0.0140 (16)	0.0058 (15)
C11	0.043 (2)	0.037 (2)	0.0296 (18)	−0.0039 (16)	0.0015 (15)	0.0068 (15)
C23	0.061 (3)	0.039 (2)	0.0279 (18)	0.0000 (19)	0.0018 (17)	−0.0034 (16)

### (2,2'-Bipyridine-κ2N,N')(4,4'-dimethoxy-2,2'-bipyridine-κ2N,N')palladium(II) bis(trifluoromethanesulfonate) Geometric parameters (Å, º)

**Table:**

Pd1—N2	2.022 (3)	C1—C2	1.370 (5)
Pd1—N1	2.029 (3)	C1—H1	0.9500
Pd1—N3	2.033 (3)	C13—C14	1.380 (5)
Pd1—N4	2.046 (3)	C13—H13	0.9500
S2—O6	1.433 (3)	C8—C9	1.389 (5)
S2—O8	1.438 (3)	C8—C7	1.402 (4)
S2—O7	1.440 (3)	C19—C20	1.388 (5)
S2—C24	1.824 (4)	C19—C18	1.391 (5)
S1—O5	1.431 (3)	C19—H19	0.9500
S1—O4	1.436 (3)	C4—C3	1.390 (5)
S1—O3	1.445 (2)	C4—H4	0.9500
S1—C23	1.814 (4)	C6—C7	1.377 (4)
F5—C24	1.314 (5)	C9—C10	1.372 (5)
O2—C8	1.336 (4)	C9—H9	0.9500
O2—C12	1.451 (4)	C10—H10	0.9500
F6—C24	1.335 (4)	C22—C21	1.391 (5)
F4—C24	1.328 (4)	C22—H22	0.9500
F1—C23	1.347 (5)	C14—C15	1.380 (5)
O1—C3	1.338 (4)	C14—H14	0.9500
O1—C11	1.442 (5)	C15—C16	1.383 (5)
F3—C23	1.333 (4)	C15—H15	0.9500
N1—C1	1.345 (4)	C3—C2	1.396 (5)
N1—C5	1.362 (4)	C16—H16	0.9500
N3—C13	1.342 (4)	C20—C21	1.376 (5)
N3—C17	1.362 (4)	C20—H20	0.9500
F2—C23	1.335 (5)	C7—H7	0.9500
N4—C22	1.338 (4)	C2—H2	0.9500
N4—C18	1.358 (4)	C21—H21	0.9500
N2—C10	1.338 (4)	C12—H12A	0.9800
N2—C6	1.361 (4)	C12—H12B	0.9800
C5—C4	1.380 (4)	C12—H12C	0.9800
C5—C6	1.476 (4)	C11—H11A	0.9800
C17—C16	1.382 (5)	C11—H11B	0.9800
C17—C18	1.465 (4)	C11—H11C	0.9800
			
N2—Pd1—N1	79.63 (10)	C8—C9—H9	120.9
N2—Pd1—N3	165.80 (10)	N2—C10—C9	123.1 (3)
N1—Pd1—N3	102.93 (10)	N2—C10—H10	118.4
N2—Pd1—N4	101.76 (10)	C9—C10—H10	118.4
N1—Pd1—N4	162.05 (10)	N4—C22—C21	122.2 (3)
N3—Pd1—N4	80.18 (10)	N4—C22—H22	118.9
O6—S2—O8	115.34 (18)	C21—C22—H22	118.9
O6—S2—O7	114.46 (17)	C15—C14—C13	119.0 (3)
O8—S2—O7	114.52 (18)	C15—C14—H14	120.5
O6—S2—C24	103.93 (17)	C13—C14—H14	120.5
O8—S2—C24	102.87 (17)	C14—C15—C16	119.1 (3)
O7—S2—C24	103.50 (18)	C14—C15—H15	120.5
O5—S1—O4	115.52 (17)	C16—C15—H15	120.5
O5—S1—O3	115.24 (16)	N4—C18—C19	121.0 (3)
O4—S1—O3	114.33 (16)	N4—C18—C17	115.2 (3)
O5—S1—C23	103.9 (2)	C19—C18—C17	123.7 (3)
O4—S1—C23	102.74 (18)	O1—C3—C4	125.5 (3)
O3—S1—C23	102.63 (17)	O1—C3—C2	115.9 (3)
C8—O2—C12	116.6 (3)	C4—C3—C2	118.6 (3)
C3—O1—C11	117.7 (3)	C17—C16—C15	119.6 (3)
C1—N1—C5	118.0 (3)	C17—C16—H16	120.2
C1—N1—Pd1	125.0 (2)	C15—C16—H16	120.2
C5—N1—Pd1	115.6 (2)	C21—C20—C19	119.1 (3)
C13—N3—C17	118.8 (3)	C21—C20—H20	120.4
C13—N3—Pd1	125.4 (2)	C19—C20—H20	120.4
C17—N3—Pd1	114.7 (2)	C6—C7—C8	118.8 (3)
C22—N4—C18	119.1 (3)	C6—C7—H7	120.6
C22—N4—Pd1	125.8 (2)	C8—C7—H7	120.6
C18—N4—Pd1	114.2 (2)	C1—C2—C3	119.4 (3)
C10—N2—C6	118.7 (3)	C1—C2—H2	120.3
C10—N2—Pd1	124.8 (2)	C3—C2—H2	120.3
C6—N2—Pd1	116.0 (2)	F5—C24—F4	108.3 (3)
N1—C5—C4	122.4 (3)	F5—C24—F6	106.5 (3)
N1—C5—C6	114.1 (3)	F4—C24—F6	106.5 (3)
C4—C5—C6	123.4 (3)	F5—C24—S2	112.6 (3)
N3—C17—C16	121.0 (3)	F4—C24—S2	111.8 (3)
N3—C17—C18	115.0 (3)	F6—C24—S2	110.8 (3)
C16—C17—C18	124.0 (3)	C20—C21—C22	119.0 (3)
N1—C1—C2	122.7 (3)	C20—C21—H21	120.5
N1—C1—H1	118.7	C22—C21—H21	120.5
C2—C1—H1	118.7	O2—C12—H12A	109.5
N3—C13—C14	122.4 (3)	O2—C12—H12B	109.5
N3—C13—H13	118.8	H12A—C12—H12B	109.5
C14—C13—H13	118.8	O2—C12—H12C	109.5
O2—C8—C9	124.1 (3)	H12A—C12—H12C	109.5
O2—C8—C7	116.7 (3)	H12B—C12—H12C	109.5
C9—C8—C7	119.2 (3)	O1—C11—H11A	109.5
C20—C19—C18	119.4 (3)	O1—C11—H11B	109.5
C20—C19—H19	120.3	H11A—C11—H11B	109.5
C18—C19—H19	120.3	O1—C11—H11C	109.5
C5—C4—C3	118.8 (3)	H11A—C11—H11C	109.5
C5—C4—H4	120.6	H11B—C11—H11C	109.5
C3—C4—H4	120.6	F3—C23—F2	107.8 (3)
N2—C6—C7	121.5 (3)	F3—C23—F1	107.9 (4)
N2—C6—C5	114.2 (3)	F2—C23—F1	106.7 (4)
C7—C6—C5	124.3 (3)	F3—C23—S1	111.1 (3)
C10—C9—C8	118.3 (3)	F2—C23—S1	111.7 (3)
C10—C9—H9	120.9	F1—C23—S1	111.4 (3)
